# Influence of Aging Treatment and Volume Fraction on Nano-Indentation Behavior of Ni-Based Single Crystal Superalloys

**DOI:** 10.3390/ma17246216

**Published:** 2024-12-19

**Authors:** Shunyong Zhang, Bin Zhang, Fengpeng Zhao, Jicheng Li, Liming Wei, Xicheng Huang

**Affiliations:** 1 Institute of Systems Engineering, China Academy of Engineering Physics, Mianyang 621999, China; zhangshunyong20@gscaep.ac.cn (S.Z.);; 2 Shock and Vibration of Engineering Materials and Structures Key Laboratory of Sichuan Province, Mianyang 621999, China

**Keywords:** Ni-based single crystal superalloys, aging treatment, nano-indentation tests, mechanical properties, mechanism analysis

## Abstract

The effects of aging treatment and the volume fraction of precipitation particles on the nano-hardness and nano-indentation morphology of Ni-based single crystal superalloys are systematically investigated. Using nano-indentation tests and atomic force microscopy (AFM), this study examined the mechanical properties and related physical mechanisms of Ni-based superalloys that have two volume fractions of precipitation particles and four aging treatment times. Results analyzed using the Oliver–Pharr method indicate that prolonging the aging time or increasing the volume fraction of particles enhances the nano-hardness and creep resistance of Ni-based single crystal superalloys and reduces the indentation-affected area. Additionally, the nano-hardness and elastic modulus decrease gradually with increasing applied force, revealing an obvious indentation size effect. These variations are closely linked to the size and density of particles and work hardening rate, as well as to the topologically close-packed (TCP) phases, which influence dislocation movement and accumulation within the material and lead to various nano-indentation behavior in Ni-based single crystal superalloys. The related study provides theoretical guidance and experimental data to support the design and application of superalloys.

## 1. Introduction

Ni-based superalloys hold a unique and important position within the superalloys field. Their exceptional mechanical properties, combined with excellent oxidation and corrosion resistance, as well as superior creep and fatigue performance at high temperatures, mean that Ni-based superalloys are widely used in the manufacturing of hot-end components for aerospace engines, gas turbines, etc. [1,2,3,4,5] However, these alloys are often subjected to complex and harsh loading conditions during service, which may cause the precipitation particles to coarsen (known as Ostwald ripening) [6,7,8]. This microstructural evolution can negatively affect the mechanical properties of alloys, posing potential safety risks to the hot-end components [9,10,11,12]. Consequently, it is essential to investigate the relationship between the mechanical properties of Ni-based superalloys and their microstructural evolution to ensure the reliability and performance of components.

Ni-based single crystal superalloys, known as two-phase alloys, are primarily strengthened by the γ′ precipitation phase-ordered L12 structure [13,14]. During aging treatment, numerous dislocations accumulate at the interface between the γ and γ′ phases, forming a dislocation network that effectively impedes dislocation movements [15,16,17,18,19,20]. After phase coarsening, the particle shape, size, and distribution change significantly; this could alter the interactions between dislocations, thereby affecting the mechanical properties of alloys [21,22,23,24,25,26,27]. Simultaneously, experimental studies also demonstrate that aging treatment will lead to significant variations in the creep, strength, fracture toughness, and ductility of materials, with different deformation and failure characteristics [20,26,28,29,30,31,32,33]. However, classical test methods provide only macroscopic mechanical properties and are usually limited to the specimen’s surface. These methods fall short in quantifying the evolution of microscopic mechanical properties under complex loading conditions and their microstructural interactions. This highlights the pressing need for advanced testing techniques capable of capturing microscopic mechanical properties.

In recent years, nano-indentation technology has been widely used in the study of metallic materials, becoming a vital tool for exploring micro-scale deformation behavior, damage processes, and property evolution [34,35,36]. Numerous studies have utilized nano-indentation tests to investigate the mechanical properties of Ni-based superalloys, e.g., hardness, yield strength, elastic modulus, etc. [37,38,39,40,41,42]. While existing nano-indentation studies primarily focus on the broad trends in mechanical property changes under specific conditions or alloy systems, less work has been carried out on systematically linking these behaviors to microstructural evolution. For example, Wu et al. [43] developed a model of precipitation strengthening in Ni-based superalloys with cuboidal γ′ particles and emphasized the critical role of multi-scale strengthening factors (including volume fraction, particle geometry, etc.). Kim et al. [44] investigated the creep behaviors of Ni-based superalloy CM 247 LC under various temperature and stress conditions, finding that secondary γ′ precipitation, eutectic γ/γ′ phases, and γ′ rafting significantly influence creep properties in Ni-based superalloys under various heat treatments. Additionally, Ruzic et al. [45] examined the temperature-dependent deformation mechanisms of γ and γ′ single-phase Ni-based superalloys, emphasizing the different roles of each phase in high-temperature nano-mechanical responses. However, systematic analysis of the relationship between nano-indentation behavior and microstructure is still lacking, especially in comparative studies of alloys with different volume fractions and aging treatment.

In this study, nano-indentation tests were conducted on Ni-based single crystal superalloys with two different volume fractions of γ′ phase (30% and 60%) and corresponding to four aging treatment times (0 h, 100 h, 500 h, and 1000 h). Atomic force microscopy (AFM) was employed to characterize the nano-indentation morphology. Additionally, the experimental results were further analyzed using the Oliver–Pharr (O&P) method, which systematically analyzes the influence of volume fraction and aging treatment on the nano-hardness and nano-indentation morphology at room temperature. Related testing and analysis provide theoretical guidance for improving the hardness of Ni-based superalloys and optimizing the design of hot-end components.

## 2. Materials and Methods

### 2.1. Materials Fabrication

To compare and analyze the influence of precipitation particles and aging treatment on the mechanical properties of Ni-based single crystal superalloys, this study selected materials with two volume fractions of precipitation particles (i.e., φ = 30%, 60%) combined with four aging treatment times (i.e., t = 0 h, 100 h, 500 h, 1000 h). To facilitate subsequent analyses, the alloys with φ = 30% were named sequentially LT-0, LT-100, LT-500, and LT-1000 alloys according to the increasing aging treatment time, and the alloys with φ = 60% were named HT-0, HT-100, HT-500, and HT-1000 alloys in the same order.

Ni-based single crystal superalloys were prepared using high rate solidification (HRS). The chemical compositions of alloys with different volume fractions are listed in Table 1. The preparation process primarily involved induction melting, casting, and directional solidification of the base material. During the process, the equipment was heated using a dual-zone graphite resistance system, with multilayer carbon felt used as a thermal radiation shield to achieve temperature gradient and precise control. In addition, the crystal selection method was employed to obtain single crystals with [001] orientation, where the start height of the helical selector was set to 40 cm, and the selection section consisted of 2.5 helical turns with a height of 30 cm. Finally, the dimensions of the prepared single crystal rods were 20 mm in diameter and 200 mm in height.

**Figure 1:**
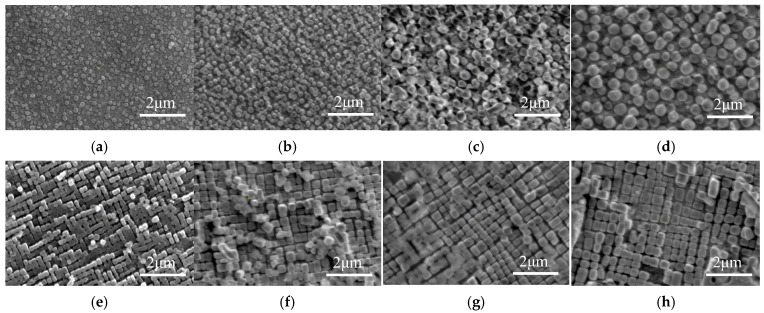
The γ/γ ′ microstructures in Ni-based single crystal superalloys with different aging times and volume fractions. (**a**) LT-0 alloy; (**b**) LT-100 alloy; (**c**) LT-500 alloy; (**d**) LT-1000 alloy; (**e**) HT-0 alloy; (**f**) HT-0 alloy; (**g**) HT-500 alloy; (**h**) HT-1000 alloy.

Heat treatment of samples was carried out in a KSL-1400X box furnace (SiC), manufactured by KJ Group of Hefei, China. The single crystal rods were placed in a uniform temperature zone for aging at 900 °C. Different specimens were taken out from furnace after 100 h, 500 h, and 1000 h treatment, respectively. The microstructures of various Ni-based single crystal superalloys are shown in Figure 1, where the coupled interface between the γ and γ′ phases is clearly visible.

### 2.2. Nano-Indentation Tests

**Figure 2:**
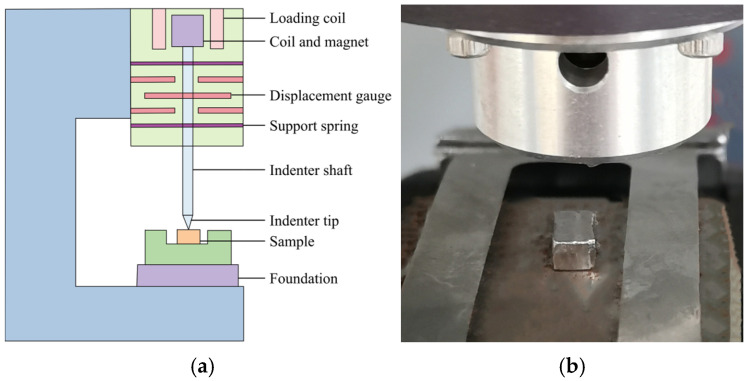
Schematic illustration and equipment of the nano-indentation system. (**a**) Schematic; (**b**) Equipment.

The nano-indentation test is widely used to evaluate the mechanical properties of materials under small loads, shallow indentation depths, and at micro-scale. This test uses a nano-indenter with excellent force and displacement resolution to continuously record the force-depth (*P-h*) curve during the loading process. Then, the loading and unloading curves are mathematically analyzed to obtain key mechanical properties, e.g., nano-hardness and elastic modulus. The nano-indentation system is shown in Figure 2.

The experiments were carried out on a nano-indenter equipped under cyclic loading conditions, with an applied force ranging from 50 mN to 400 mN. The equipment has a load resolution of 50 nN and a displacement resolution of 0.01 mm. The loading procedure was as follows: the loading rate/loading force (*P/P*) was kept constant under load control mode until the preset load was reached. The load was then held at the maximum for 5 s to minimize creep effects [46]. Afterward, the load was reduced at the same rate to 10% of the maximum, where it was held for another 5 s to eliminate thermal drift effects, before being fully unloaded. For each sample, 16 indentation points were arranged in a 4 × 4 array, with a spacing of over 0.1 mm between points to ensure independent and reliable test results [47].

### 2.3. Nano-Indentation Response and Property

**Figure 3:**
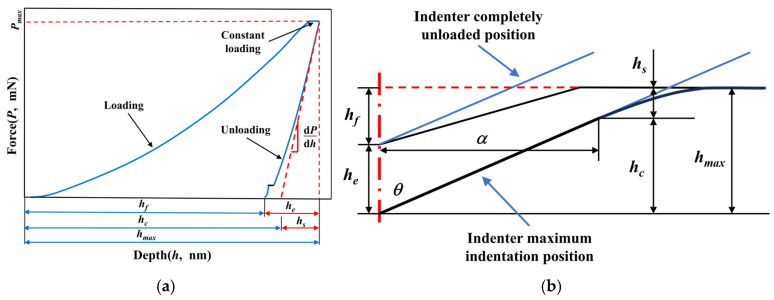
Typical *P-h* curve and schematic of indentation profile. (**a**) Typical *P-h* curve for nano-indentation test; (**b**) Schematic of indentation profile.

Figure 3a shows the typical force (*P*)-depth (*h*) curve in the nano-indentation test, and Figure 3b further illustrates a detailed cross-sectional profile of the indentation at the maximum depth and after complete unloading [38,39,48,49,50]. Using the O&P method [51,52], the nano-hardness and elastic modulus of materials could be calculated from the unloading part of the *P-h* curve. Furthermore, numerous experimental data indicate that the *P-h* curve can be described with a power-law equation, as follows:

(1) $$P=\alpha {\left(h-h_{f}\right)}^{m},$$

where $h_{f}$ represents the residual indentation depth, while α and m are empirical constants determined through curve fitting. The slope of the unloading curve at the maximum indentation depth ($h_{max}$) is defined as the contact stiffness, which can be calculated as below:

(2) $$S={\left(\frac{dP}{dh}\right)}_{h=h_{max}}=\alpha m{\left(h_{max}-h_{f}\right)}^{m-1}.$$

According to the definition of elastic modulus and the assumption that dislocation pile-up is negligible in nano-indentation tests [52], the sink-in depth ($h_{s}$) and contact depth ($h_{c}$) can be calculated using the following equations:

(3) $$h_{s}=\omega \frac{P_{max}}{S}$$

(4) $$h_{c}=h_{max}-h_{s}=h_{max}-\omega \frac{P_{max}}{S},$$

where $P_{max}$ represents the peak force and ω is a constant depending on indenter geometry (for the Berkovich indenter, ω = 0.75). Additionally, the relationship between the projected area ($A_{c}$) and the contact depth is as follows:

(5) $$A_{c}=f\left(h_{c}\right)=3\sqrt{3}\tan^{2}\left(\alpha \right)h_{c}^{2}=\xi h_{c}^{2},$$

where α is the half-angle of the indenter, which is approximately 65.3° for the Berkovich indenter, i.e., ξ = 24.56. By substituting the peak force and projected area into Equations (6) and (7), the nano-hardness (H) and reduced modulus ($E_{r}$) of the tested material can be directly calculated [52]:

(6) $$H=\frac{P_{max}}{A_{c}}$$

(7) $$E_{r}=\frac{\sqrt{\pi }S}{2\beta \sqrt{A_{c}}},$$

where β denotes the indenter effect in terms of geometrical constant (for the Berkovich indenter, β = 1.034). When the values of the elastic modulus ($E_{i}$) and Poisson’s ratio ($\nu_{i}$) of the indenter (for the diamond, $E_{i}$ = 1140 GPa, $\nu_{i}$ = 0.07) are known, the elastic modulus (E) of tested material can be derived:

(8) $$\frac{1}{E_{r}}=\frac{1-\nu_{i}^{2}}{E_{i}}+\frac{1-\nu^{2}}{E},$$

where ν represents the Poisson’s ratio of tested material; ν = 0.3 for Ni-based superalloys.

### 2.4. Nano-Indentation Morphology and Characterization

AFM is a high-resolution analytical tool that is widely used to investigate the surface topography of solid materials with atomic-scale precision. AFM typically operates in two modes: contact mode and tapping mode.

**Figure 4:**
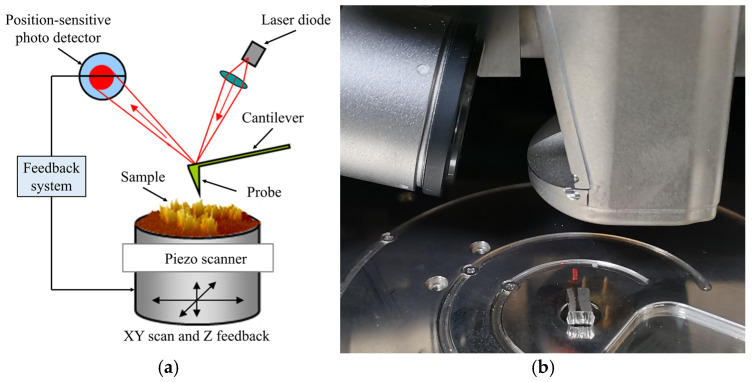
Schematic illustration and equipment of tapping mode AFM. (**a**) Schematic; (**b**) Equipment.

In this study, tapping mode AFM was employed to perform high-precision imaging of the indentation surface. The core principle of this technique relies on the interaction between the oscillating cantilever and the sample surface. As the tip approaches the surface, atomic forces cause slight variations in the oscillation amplitude of cantilever. By tracking these amplitude changes, a detailed two-dimensional profile of the nano-indentation can be generated. The corresponding tapping mode AFM is shown in Figure 4.

## 3. Results and Analysis

### 3.1. Force-Depth Curves

**Figure 5:**
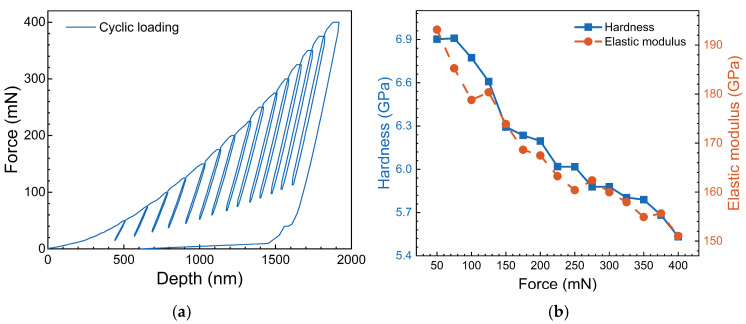
The performance parameters of LT-0 alloy for cyclic loading in nano-indentation tests. (**a**) *P-h* curve for cyclic loading; (**b**) Hardness and elastic models for cyclic loading.

A cyclic nano-indentation test was conducted on the LT-0 alloy, with peak force increments ranging from 50 to 400 mN at 25 mN intervals; the force-depth (*P-h*) curves are shown in Figure 5a. Based on these curves and the relevant equations presented in Section 2.3, the nano-hardness and elastic modulus of alloys under cyclic loading conditions were calculated, as illustrated in Figure 5b. The results demonstrate that both nano-hardness and elastic modulus follow similar trends, decreasing similarly to the peak force. This behavior can be attributed to the indentation size effect commonly observed in nano-indentation tests [49,53,54,55]. To accurately assess the influence of aging treatment or volume fraction on the mechanical properties of Ni-based single crystal superalloys, consistent indentation depths or peak force should be maintained among all tests. It can also be seen from Figure 5b that at a lower peak force, the decrease in nano-hardness and elastic modulus is more pronounced, whereas as the peak force increases, this trend gradually stabilizes, aligning with other nano-indentation test results [54,56,57,58,59].

**Figure 6:**
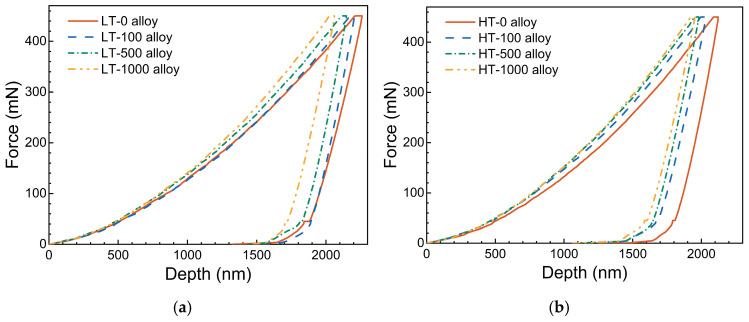
*P-h* curves corresponding to different alloys in nano-indentation tests. (**a**) φ = 30%; (**b**) φ = 60%.

Figure 6 further illustrates the *P-h* curves from nano-indentation tests conducted on various Ni-based single crystal superalloys, where the peak force is set at 450 mN and the indentation depth reaches approximately 2 μm. The curves show that the *P-h* curve features among the different alloys are quite similar. As the indenter displacement increases, the force gradually rises, with the rate of rise accelerating. During the hold period at peak force, the indentation depth increases slowly. Upon unloading, the load gradually decreases as the indenter retracts.

In addition, it is also found from Figure 6 that the alloys without aging treatment (i.e., LT-0 and HT-0 alloy) exhibit greater indentation depths under the same force, indicating more significant plastic deformation. As the aging time increases, the maximum indentation depth decreases, suggesting significant hardening due to aging treatment. Additionally, comparing Figure 6a,b, the maximum indentation depth decreases noticeably as the volume fraction of particles increases, reflecting the strengthening effect of precipitation particles in the alloys. It is worth noting that the unloading part of most alloys shows similar trends. However, the *P-h* curve of LT-0 alloy decreases more slowly during unloading, and under the same force, its indenter displacement becomes smaller than that of the LT-100 alloy. This indicates that the LT-0 alloy has better ductility but relatively low nano-hardness.

It is worth noting that creep resistance is positively correlated with the nano-hardness of the material. As the aging time increases, the indenter displacement during the holding phase gradually decreases. This indicates that the creep resistance of the alloy increases relatively after aging treatment. In addition, the indenter displacement during the holding phase reduces along with the volume fraction of particles, signifying that creep resistance enhances with the increasing volume fraction of particles. These findings highlight the strong dependence of creep resistance on both aging treatment and volume fraction.

### 3.2. Mechanical Properties

Based on the nano-indentation response equations outlined in Section 2.3 and the *P-h* curves obtained for different alloys under 450 mN, the mechanical properties, including nano-hardness and elastic modulus, are calculated and listed in Table 2. It can be seen that there are significant differences in nano-hardness among different alloys, while the elastic modulus remains consistently around 141 GPa at room temperature, i.e., the volume fraction and aging treatment have minimal influence on the elastic modulus.

**Figure 7:**
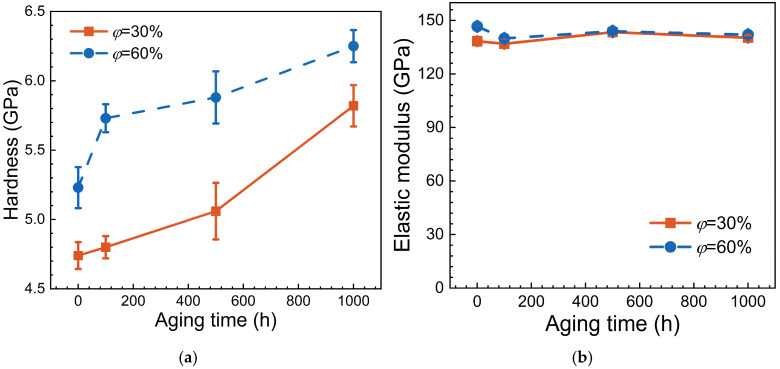
Hardness and elastic modulus corresponding to different alloys in nano-indentation tests. (**a**) Hardness; (**b**) Elastic modulus.

In the following, the variation of nano-hardness and elastic modulus with aging time is shown in Figure 7, respectively. It can be observed that as the volume fraction of particles increases, the nano-hardness of alloys increases significantly, i.e., precipitation particles play a strengthening role in the nano-hardness of Ni-based single crystal superalloys. Additionally, with the increase of aging time, the nano-hardness increases monotonically, indicating significant hardening after aging treatment. It is worth noting that the variation trend of nano-hardness differs for alloys with different volume fractions. Generally, in the early stage of aging treatment (i.e., t = 0~100 h), the alloys with a higher volume fraction show a more pronounced increase in nano-hardness. However, in the middle and later stages of aging treatment (i.e., t = 100~1000 h), the alloys with a lower volume fraction experience a larger increase in nano-hardness. This suggests that the influence of particles enhances initially but then gradually weakens along with aging time.

It can be seen from Figure 7b that the elastic modulus of nickel-based single crystal superalloys exhibits minimal variation along with aging time, fluctuating around 141 GPa with an error range within ± 3%, which may be due to experimental measurement errors. The data also suggest that alloys with higher volume fractions tend to have a slightly higher elastic modulus. This implies that the particles themselves may have a relatively high elastic modulus.

### 3.3. Nano-Indentation Morphology

**Figure 8:**
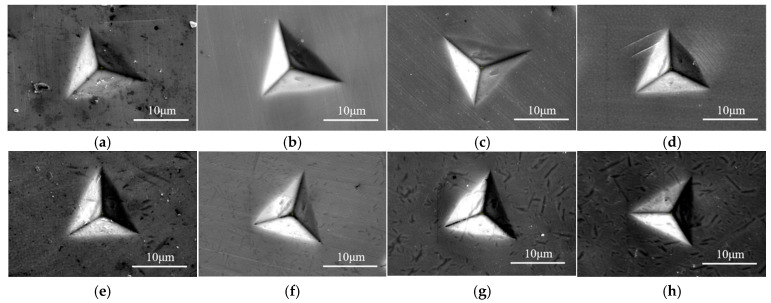
SEM morphologies corresponding to different alloy surfaces in nano-indentation tests. (**a**) LT-0 alloy; (**b**) LT-100 alloy; (**c**) LT-500 alloy; (**d**) LT-1000 alloy; (**e**) HT-0 alloy; (**f**) HT-100 alloy; (**g**) HT-500 alloy; (**h**) HT-1000 alloy.

Figure 8 demonstrates the SEM images of nano-indentation for different Ni-based single crystal superalloys. It can be observed that for the alloys with φ = 30%, samples aged for 0 h, 100 h, and 500 h (i.e., LT-0, LT-100, and LT-500 alloys) exhibit uniform pile-ups around the nano-indentations, without shear slip bands, as shown in Figure 8a–c. This suggests that the material mainly absorbs and dissipates stress through surface pile-up, and plastic deformation is concentrated near the contact surfaces. However, in the sample aged for 1000 h (i.e., LT-1000 alloy), clear shear slip bands are observed around the indentation, indicating the onset of plastic flow, as shown in Figure 8d. The formation of these slip bands indicates a significant increase in dislocation density, thereby increasing the nano-hardness of alloys. Furthermore, visible cracks around the nano-indentation of the LT-1000 alloy indicate reduced crack resistance, i.e., the alloy exhibits brittle behavior and low fracture toughness under localized loading conditions. This explains the marked increase in nano-hardness after aging treatment (see Figure 7a).

For the alloys with φ = 60%, uniform pile-ups are observed in all samples, with no noticeable shear slip bands or cracks, as shown in Figure 8e–h. This indicates that the material primarily resists deformation through surface pile-up rather than significant plastic deformation. Additionally, SEM images of non-electrolytic corrosion demonstrate the precipitation of numerous new particles around the indents in samples from later aging treatment, and this also contributes to the hardness of alloys.

**Figure 9:**
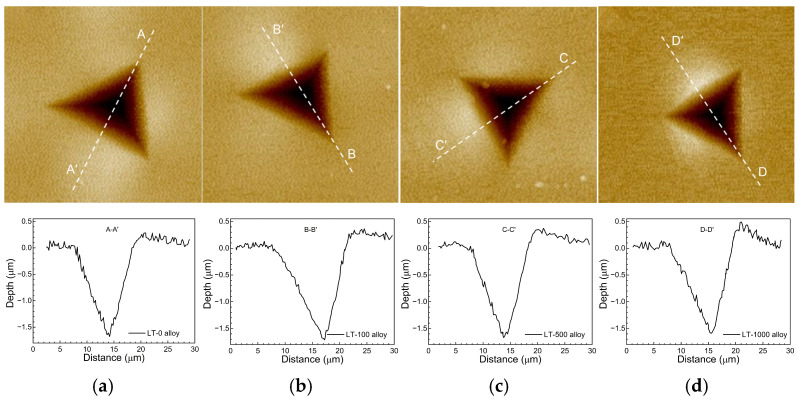
AFM images and surface profiles of indentations for the alloys with φ = 30%. (**a**) LT-0 alloy; (**b**) LT-100 alloy; (**c**) LT-500 alloy; (**d**) LT-1000 alloy.

**Figure 10:**
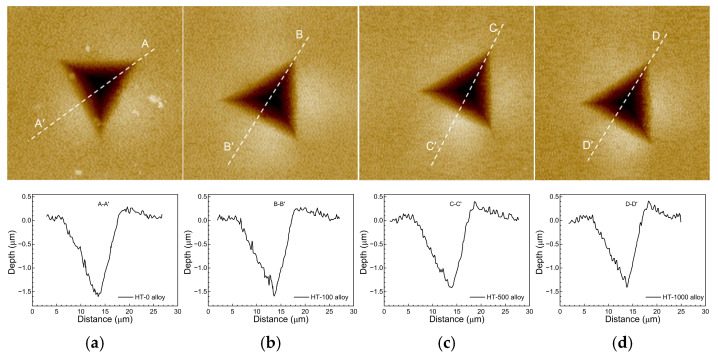
AFM images and surface profiles of indentations for the alloys with φ = 60%. (**a**) HT-0 alloy; (**b**) HT-100 alloy; (**c**) HT-500 alloy; (**d**) HT-1000 alloy.

Figure 9 and Figure 10 display the AFM images and surface profiles of indentations for the different Ni-based single crystal superalloys. It can be observed that the indentation shapes for all samples exhibit a “V” pattern, with the left side (near the vertex) showing greater fluctuations in the profile curve, indicating a greater roughness. In addition, the pile-up area is mainly concentrated in the middle regions of the indentation edges, with relatively low heights at the vertex. It can also be seen from Figure 9 and Figure 10 that with the increase of aging time, the maximum height of pile-up areas increases, while both indentation depth and pile-up volume decrease. This suggests that coarsened particles make the material more resistant to plastic deformation. It is worth noting that the indentation depth reduction trend is more significant with higher volume fractions, i.e., the influence of aging treatment becomes more pronounced as the volume fraction increases. Therefore, the indentation profile is closely related to the size and density of particles.

**Figure 11:**
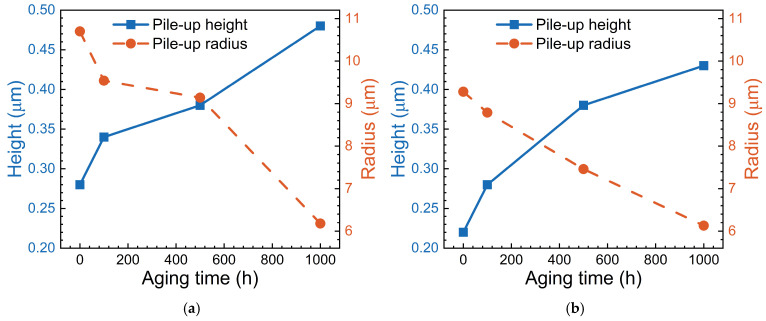
Pile-up height and pile-up radius of indentations for different alloys. (**a**) φ = 30%; (**b**) φ = 60%.

Figure 11 quantitatively characterizes the pile-up height and radius of indentations from Figure 9 and Figure 10. It can be observed that as the aging time increases, the pile-up height around the indentation also increases while the pile-up radius gradually decreases. This indicates that after aging treatment, the hardness of alloys gradually increases and the influence range of indentation becomes more localized. Figure 11 also shows that as the volume fraction of particles increases, both the pile-up height and radius significantly decrease, i.e., precipitation particles weaken the deformation caused by the indentation. It should be noted that the nano-hardness is directly proportional to the pile-up height and inversely proportional to the pile-up radius. Therefore, the nano-indentation behavior of Ni-based single crystal superalloys is closely related to both the volume fraction and aging treatment.

In addition, the pile-up on the indentation surface significantly affects the accuracy of mechanical property testing. The shrinkage behavior is usually described using the depth ratio ($h_{f}/h_{max}$) at unloading and peak force. For various materials, when 0.8 < $h_{f}/h_{max}$ ≤ 1, the contact area calculated using the O&P method is relatively accurate for materials that tend to exhibit work hardening. In contrast, for materials that do not easily work harden, the calculated values tend to be low. When $h_{f}/h_{max}$ ≤ 0.8, the O&P method is applicable to various materials, and the corresponding calculations yield reliable results [60,61].

**Figure 12:**
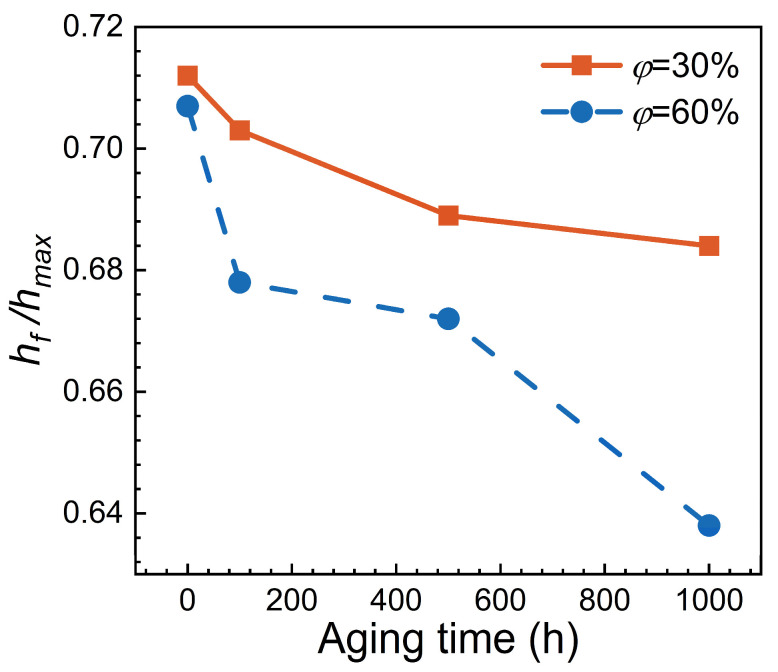
Curves of *h_f_/h_max_* ratio for different alloys in nano-indentation test.

Figure 12 shows the $h_{f}/h_{max}$ ratios for different samples, where the $h_{f}/h_{max}$ ratios are all below 0.8, which means the nano-indentation tests conducted in this study can accurately calculate the nano-hardness of Ni-based single crystal superalloys, and the influence of pile-up on the mechanical property assessments is negligible. Furthermore, Figure 12 also illustrates that as aging time or volume fraction increases, the $h_{f}/h_{max}$ ratios of samples gradually decrease, i.e., the rebound magnitude during the unloading part increases. This implies that the alloys experience relatively less plastic deformation, which contributes to the nano-hardness of alloys.

## 4. Mechanism Analysis Mechanism Analysis

In the following, the mechanisms by which the volume fraction of precipitation particles and aging treatment influence the nano-hardness of Ni-based single crystal superalloys are analyzed. For precipitation-hardening alloys, the precipitation particles act as a series of point barriers that interact with dislocations and impede their movement [62,63]. Dislocations overcome the hindrance posed by particles primarily through two mechanisms: cutting through particles and bypassing particles. Consequently, there exists a critical size for the precipitation particles at which the deformation mechanism transforms from dislocation cutting to bypassing [15]. When the particles are smaller than the critical size, the dislocation-cutting mechanism predominates. This means that as the size of precipitation particles increases, the shear stress required for dislocations to cut through them also increases, thereby enhancing the precipitation-strengthening effect [64]. When the particles exceed the critical size, the primary deformation mechanism of dislocation transforms from direct cutting to bypassing the particles, with the bypass mechanism taking precedence. According to the Orowan mechanism, it is known that as the distribution of particles becomes sparser, the precipitation-strengthening effect gradually weakens [65,66]. Thus, in the early stage of aging treatment, the size and volume fraction of particles are positively correlated with the hardness. However, once coarsening reaches a certain extent, the hardness gradually decreases along with aging time.

As shown in the microstructure images of alloys in Figure 1, when the aging treatment reaches 1000 h, no dislocation bypass channels are observed in the matrix phase. This indicates that the particle size remains smaller than the critical size, and the dislocation-cutting mechanism is still dominant. Therefore, as the aging time increases, the size of particles within the alloy gradually grows, and this leads to an increase in the shear stress required for dislocations to pass through the particle, i.e., the nano-hardness of alloys gradually increases. Furthermore, from Figure 1 it can also be found that the number and density of particles in the alloy with φ = 60% are significantly higher than those in the alloy with φ = 30%, thereby enhancing the obstructive effect on dislocation movement, which leads to a relatively higher hardness of the alloy with φ = 60%. More detailed and specific dislocation movement and the interaction between dislocations and γ′ precipitation were studied in our previous work [23,24,25]. Hence, the hardness increases with the increasing volume fraction of particles.

**Figure 13:**
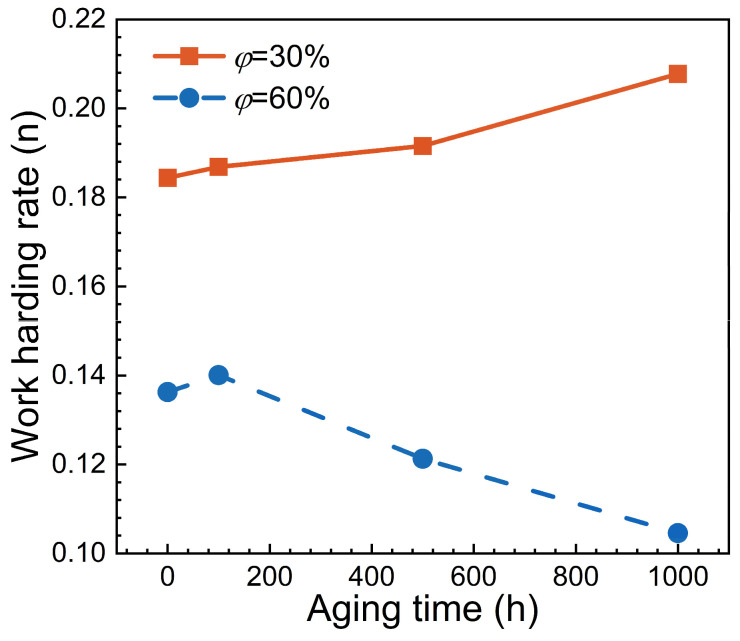
Work hardening rate of the Ni-based superalloys with different aging times.

Plastic deformation in Ni-based superalloys is primarily driven by dislocation movement, and dislocation density generally exhibits a positive correlation with plastic strain. Dislocation motion is mainly hindered by grain boundaries, causing dislocation pile-up at these areas, and thus, the resistance for plastic deformation is enhanced, i.e., the alloy produces work hardening [1]. Consequently, the hardness of materials is closely related to their work hardening rate. The work hardening rate curves, which are obtained from quasi-static tensile tests conducted by the authors’ group on Ni-based single crystal superalloys, are shown in Figure 13. It can be observed that for the alloys with φ=30%, the work hardening rate increases along with aging time, and is consistent with the variation characteristics of nano-hardness, effectively explaining the physical mechanism of the gradual increase in nano-hardness. Figure 13 also shows that during the early and middle stages of aging treatment (i.e., t = 0~500 h), the work hardening rate increases only slightly, whereas it significantly rises during the later aging stage (i.e., t = 500~1000 h), and this coincides well with the variation features of nano-hardness.

**Figure 14:**
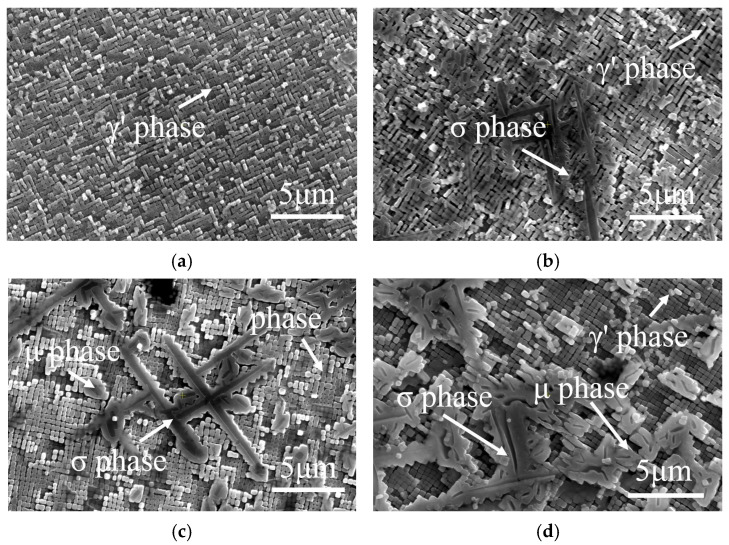
SEM image of TCP phase in the alloys with φ = 60%. (**a**) HT-0 alloy; (**b**) HT-100 alloy; (**c**) HT-500 alloy; (**d**) HT-1000 alloy.

In contrast, for the alloys with φ = 60, the trends in hardness and work hardening rate are not entirely consistent. For example, during the middle and later stages of aging treatment (i.e., t = 100~1000 h), the work hardening rate gradually decreases, while hardness continues to rise (see Figure 7). This difference is closely linked to the extensive precipitation of harmful TCP phases within the alloy. After aging treatment, the hard and brittle needle-like σ phases and rhombohedral μ phases are observed within the material, as shown in Figure 14. In addition, backscattered electron (BSE) imaging and X-ray diffraction (XRD) were utilized to further analyze the phase composition at specific regions in the SEM micrographs. It can be seen that the amount of TCP phase within the matrix increases with increasing aging time, thereby contributing to the gradual rise in alloy hardness. Therefore, it can be concluded that there are several factors influencing the nano-hardness of the alloy, including work hardening rate and TCP phase, etc.

## 5. Conclusions

This study systematically investigated the influence of aging treatment and volume fraction on the nano-indentation behavior of Ni-based single crystal superalloys. Combining nano-indentation tests, AFM characterization, and dislocation movement mechanisms, this work provides a comprehensive understanding of the relationship between microstructural evolution and nano-mechanical properties. The main conclusions can be summarized as follows:

(1) In the nano-indentation tests, as the volume fraction or aging time increases, the nano-hardness of Ni-based single crystal superalloys gradually increases, exhibiting a complex variation trend. In contrast, the elastic modulus shows minimal variation among different samples, indicating minimal sensitivity to microstructural evolution.
(2) Aging treatment and volume fraction of particles significantly affect the nano-indentation morphology. As the aging time increases, the indentation-affected area decreases, signifying reduced plastic deformation properties and more uniform microscopic deformation. Additionally, higher volume fractions or extended aging times result in lower $h_{f}/h_{max}$ ratios and greater rebound during unloading, corresponding to increased nano-hardness.
(3) The nano-indentation behavior is closely related to the microstructure of materials, especially to the size and density of precipitation particles. The microstructural evolution significantly influences the nucleation and movement of dislocations, thereby affecting the macro-mechanical properties of materials. It is worth noting that the particle sizes of the studied materials are smaller, i.e., the dislocation cutting mechanism still plays a key role. For low volume fractions (φ = 30%), wider dislocation channels reduce the hindrance of dislocation movement, leading to relatively low nano-hardness. Conversely, for the alloys with φ = 60%, the harmful TCP phases precipitate substantially within the matrix with increasing aging time, further enhancing the alloys’ nano-hardness.
(4) This work provides new insights into how microstructural evolution affects the nano-indentation behavior, such as nano-hardness, deformation uniformity, indentation-affected area, etc. In addition, a related study also offers reliable experimental data and theoretical support for investigating the performance variations of Ni-based superalloys under high-temperature and complex loading conditions.

## Figures and Tables

**Table 1:** Chemical composition of Ni-based single crystal superalloys.

φ	C	Al	Cr	Co	Ta	W	Ni
30%	0.021	4.00	18.63	13.91	3.51	4.04	Bal.
60%	0.014	6.16	18.58	9.49	5.17	4.00	Bal.

**Table 2:** Performance parameters of different alloys in nano-indentation tests.

φ (%)	Aloys	Elastic Modulus (GPa)	Nano-Hardness (GPa)
30	LT-0 alloy	138.38 ± 2.27	4.72 ± 0.09
	LT-100 alloy	136.89 ± 1.98	4.80 ± 0.08
	LT-500 alloy	143.40 ± 2.23	5.06 ± 0.20
	LT-1000 alloy	140.29 ± 1.19	5.82 ± 0.15
60	HT-0 alloy	146.65 ± 1.85	5.23 ± 0.15
	HT-100 alloy	133.87 ± 1.39	5.74 ± 0.10
	HT-500 alloy	143.95 ± 1.46	5.88 ± 0.19
	HT-1000 alloy	141.97 ± 1.57	6.25 ± 0.12

## Data Availability

The original contributions presented in the study are included in the article; further inquiries can be directed to the corresponding author.
